# Hypertension Among Youths — United States, 2001–2016

**DOI:** 10.15585/mmwr.mm6727a2

**Published:** 2018-07-13

**Authors:** Sandra L. Jackson, Zefeng Zhang, Jennifer L. Wiltz, Fleetwood Loustalot, Matthew D. Ritchey, Alyson B. Goodman, Quanhe Yang

**Affiliations:** ^1^Division for Heart Disease and Stroke Prevention, National Center for Chronic Disease Prevention and Health Promotion, CDC; ^2^United States Public Health Service; ^3^Division of Nutrition, Physical Activity, and Obesity, National Center for Chronic Disease Prevention and Health Promotion, CDC.

Hypertension is an important modifiable risk factor for cardiovascular morbidity and mortality, and hypertension in adolescents and young adults is associated with long-term negative health effects (*1*,*2*).* In 2017, the American Academy of Pediatrics (AAP) released a new Clinical Practice Guideline (*3*), which updated 2004 pediatric hypertension guidance^†^ with new thresholds and percentile references calculated from a healthy-weight population. To examine trends in youth hypertension and the impact of the new guideline on classification of hypertension status, CDC analyzed data from 12,004 participants aged 12–19 years in the 2001–2016 National Health and Nutrition Examination Survey (NHANES). During this time, prevalence of hypertension declined, using both the new (from 7.7% to 4.2%, p<0.001) and former (from 3.2% to 1.5%, p<0.001) guidelines, and declines were observed across all weight status categories. However, because of the new percentile tables and lower threshold for hypertension (*4*), application of the new guideline compared with the former guideline resulted in a weighted net estimated increase of 795,000 U.S. youths being reclassified as having hypertension using 2013–2016 data. Youths who were older, male, and those with obesity accounted for a disproportionate share of persons reclassified as having hypertension. Clinicians and public health professionals might expect to see a higher prevalence of hypertension with application of the new guideline and can use these data to inform actions to address hypertension among youths. Strategies to improve cardiovascular health include adoption of healthy eating patterns and increased physical activity (*3*).

NHANES is a nationally representative survey of noninstitutionalized persons in the United States. The survey includes an in-person examination with up to three brachial systolic blood pressure (SBP) and diastolic blood pressure (DBP) readings taken by certified examiners. Mean SBP and DBP values were used.^§^ Among 13,523 participating youths during 2001–2016, those missing SBP or DBP (999), or body mass index (BMI [kg/m^2^]) (136) were excluded. In addition, youths classified as underweight (BMI-for-age <5th percentile; 384) were excluded because of insufficient sample size, leaving 12,004 persons aged 12–19 years in the analytic sample.

Elevated blood pressure (BP) and hypertension were defined according to age-specific thresholds established in both the former and new guidelines. To apply the former guideline, among those aged 12–17 years, elevated BP (formerly “prehypertension”) was defined as BP ≥90th to <95th percentile or ≥120/80 mmHg to <95th percentile; hypertension was defined as BP ≥95th percentile (using 2004 age, sex, and height-specific percentile tables) or reported antihypertensive medication use (only available for persons aged >15 years^¶^) (Supplementary Table 1, https://stacks.cdc.gov/view/cdc/56579). Among persons aged 18–19 years, elevated BP was defined as SBP ≥120 mmHg to <140 mmHg or DBP ≥80 mmHg to <90 mmHg; hypertension was defined as BP ≥140/90 mmHg or reported antihypertensive medication use.

The new guideline used new percentile tables (from a reference population excluding youths with overweight/obesity). To apply the new guideline, among adolescents aged 12–17 years, elevated BP was defined as BP ≥90th to <95th percentile or SBP ≥120 mmHg to <95th percentile; hypertension was defined as BP ≥95th percentile, BP ≥130/80 mmHg, or reported antihypertensive medication use. For persons aged 18–19 years, elevated BP was defined as SBP ≥120 mmHg to <130 mmHg and DBP <80 mmHg; hypertension was defined as BP ≥130/80 mmHg or antihypertensive medication use. The new guideline thresholds for persons aged 18–19 years align with recommendations in the 2017 Hypertension Clinical Practice Guideline for persons aged ≥18 years.**

Weight status was categorized using age- and sex-specific reference values from the 2000 CDC growth charts^††^ (healthy weight: BMI-for-age ≥5th to <85th percentiles; overweight: ≥85th to <95th; obesity: ≥95th). In addition, a subset of the group with obesity (severe obesity, defined as BMI-for-age ≥120% of the 95th percentile) was examined (*5*). Race/ethnicity was classified as non-Hispanic white, non-Hispanic black, Mexican American, and other.^§§^

Participant characteristics across survey years were compared using Satterthwaite chi-squared tests and t-tests. Estimated prevalence of elevated BP, hypertension, and the combination of these were calculated in 4-year increments (to assure sufficient sample size) from 2001 to 2016, and trends were assessed using survey logistic regression adjusted for age, sex, and race/ethnicity. Using prevalence estimates from 2013 to 2016, population-level estimates of the number of youths classified as having hypertension were calculated. Bootstrap methodology with 1,000 resamples was used to estimate 95% confidence intervals for the percentage of the population reclassified as having hypertension. All analyses used exam sample weights and statistical procedures for complex surveys, and all tests were two-sided.

Population characteristics were mostly consistent from 2001 to 2016, although the prevalence of obesity increased from 17.8% (2001–2004) to 21.8% (2013–2016) (p = 0.016), as did the prevalence of severe obesity (5.7% to 8.8%, p = 0.003) (Table 1). During 2001–2016, the prevalence of hypertension declined, according to both the new (from 7.7% to 4.2%, p<0.001) and former (from 3.2% to 1.5%, p<0.001) guidelines (Figure) (Supplementary Table 2, https://stacks.cdc.gov/view/cdc/56580). This decline occurred across all BMI categories, although the prevalence of hypertension was consistently highest among persons with obesity and severe obesity. During 2013–2016, using the new guideline, the prevalence of elevated BP was approximately 10%, and the prevalence of combined elevated BP or hypertension was nearly 15% (Figure).

**TABLE 1:** Characteristics of youths aged 12–19 years — National Health and Nutritional Examination Survey (NHANES), United States, 2001–2016

Characteristic	% (95% CI)				P-value for trend*
	NHANES 2001–2004 (N = 4,169)	NHANES 2005–2008 (N = 3,076)	NHANES 2009–2012 (N = 2,319)	NHANES 2013–2016 (N = 2,440)	
**Age group (yrs)**					
12–17	78.0 (75.1–80.6)	77.6 (75.1–80.0)	78.1 (75.3–80.6)	78.9 (76.9–80.8)	0.539
18–19	22.0 (19.4–24.9)	22.4 (22.0–24.9)	21.9 (19.4–24.7)	21.1 (19.2–23.1)	
**Sex**					
Male	50.8 (48.9–52.7)	51.5 (49.2–53.9)	50.8 (48.2–53.4)	50.4 (48.0–52.8)	0.703
Female	49.2 (47.3–51.1)	48.5 (46.1–50.8)	49.2 (46.6–51.8)	49.6 (47.2–52.0)	
**Race**/**Ethnicity**					
White, non-Hispanic	63.2 (57.6–68.5)	61.7 (56.6–66.6)	56.5 (50.4–62.3)	54.0 (46.7–61.2)	0.024
Black, non-Hispanic	14.0 (11.2–17.4)	15.2 (11.9–19.2)	15.0 (11.5–19.4)	14.1 (10.5–18.6)	0.987
Mexican American	10.8 (8.3–14.1)	12.0 (9.6–14.8)	13.8 (10.5–17.9)	14.7 (10.9–19.4)	0.100
Other	12.0 (9.1–15.7)	11.1 (8.5–14.4)	14.7 (12.2–17.6)	17.2 (14.9–19.8)	0.004
**Weight status^†^**					
Healthy	66.0 (63.0–68.9)	64.1 (61.8–66.6)	64.0 (61.4–66.5)	59.8 (56.7–62.7)	0.005
Overweight	16.2 (14.4–18.2)	16.6 (15.1–18.2)	15.1 (13.6–16.7)	18.4 (16.7–20.3)	0.218
Obesity (all)	17.8 (15.8–19.9)	19.2 (16.8–21.9)	20.9 (18.9–23.2)	21.8 (19.0–24.9)	0.016
Severe obesity	5.7 (4.6–7.1)	6.6 (5.2–8.3)	7.5 (5.8–9.7)	8.8 (7.3–10.6)	0.003

**Abbreviations:** BMI = body mass index; CI = confidence interval.

* P-values for trends in participant characteristics across survey years were obtained using Satterthwaite chi-squared tests and t-tests. All tests were 2-tailed.

^†^ BMI is compared with age- and sex-specific reference values from the 2000 CDC growth charts (https://www.cdc.gov/growthcharts/cdc_charts.htm). Healthy = BMI-for-age ≥5th to <85th, overweight = BMI-for-age ≥85th to <95th percentile, obesity = BMI-for-age ≥95% percentile. Severe obesity = BMI-for-age ≥120% of the 95th percentile. Persons classified as underweight (BMI-for-age <5th percentile) are excluded.

**FIGURE:**
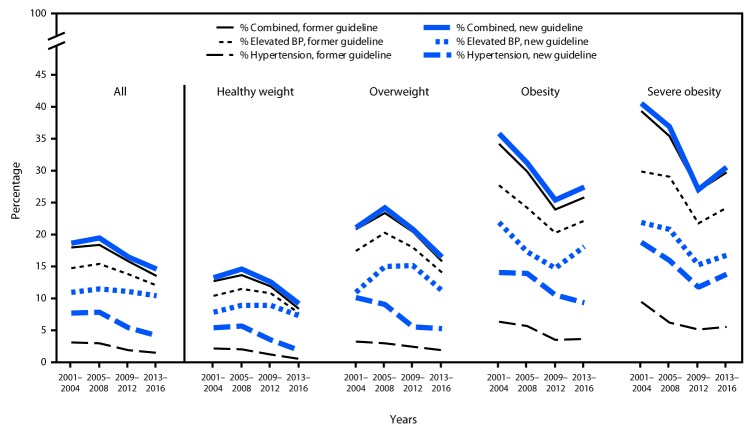
Prevalence of elevated blood pressure (BP) and hypertension among youths, by new and former guidelines — United States, 2001–2016

Compared with the former guideline, the new guideline classified fewer youths with elevated BP and more youths as having hypertension (Figure). Using data from 2013 to 2016, an additional 2.6% of U.S. youths aged 12–19 years would be reclassified as having hypertension, which translates to a net increase of approximately 795,000 persons (Table 2). Youths aged 18–19 years would account for approximately half of the net increase, and males would account for over two thirds. Nearly half of the net increase in new diagnoses of hypertension among youths would be among those with obesity (Table 2), although less than one quarter of U.S. youths have obesity (Table 1).

**TABLE 2:** Estimated hypertension prevalence and population classification by new* and former**^†^** guidelines — National Health and Nutritional Examination Survey 2013–2016

Characteristic (no.)	No. (weighted)	Estimated hypertension prevalence (new guidelines) % (95% CI)	Hypertension prevalence (former guidelines) % (95% CI)	No. of persons with hypertension (new guidelines)	No. of persons with hypertension (former guidelines)	Net increase in no. of persons with hypertension	Percentage of population reclassified as having hypertension
**All, aged 12–19 yrs (2,440)**	**30,855,000**	**4.11 (3.22–5.24)**	**1.54 (1.01–2.23)**	**1,269,000**	**474,000**	**795,000**	**2.58 (1.84–3.34)**
**Age group (yrs)**							
12–17 (1,898)	24,352,000	3.21 (2.40–4.28)	1.62 (0.97–2.52)	781,000	394,000	387,000	1.59 (0.95–2.29)
18–19 (542)	6,503,000	7.50 (5.00–10.73)	1.23 (0.48–2.56)	488,000	80,000	408,000	6.29 (3.98–8.93)
**Sex**							
Male (1,220)	15,550,000	5.78 (4.33–7.67)	2.18 (1.39–3.25)	899,000	339,000	560,000	3.62 (2.35–5.00)
Female (1,220)	15,305,000	2.42 (1.41–3.84)	0.88 (0.44–1.58)	370,000	135,000	235,000	1.53 (0.88–2.32)
**Race**/**Ethnicity**							
White, non-Hispanic (641)	16,669,000	2.97 (1.73–4.74)	0.80^¶^ (0.21–2.08)	495,000	133,000	362,000	2.17 (1.09–3.43)
Black, non-Hispanic (583)	4,345,000	6.27 (3.84–9.59)	2.94 (1.44–5.30)	273,000	128,000	145,000	3.37 (1.89–5.05)
Mexican American (549)	4,525,000	4.94 (3.01–7.59)	2.33 (1.19–4.09)	224,000	106,000	118,000	2.58 (1.29–4.04)
Other (667)	5,315,000	5.22 (3.65–7.20)	2.02 (1.09–3.40)	277,000	107,000	170,000	3.23 (1.86–4.79)
**Weight status** ^§^							
Healthy (1,423)	18,439,000	1.88 (1.12–2.97)	0.62^¶^ (0.28–1.18)	347,000	114,000	234,000	1.28 (0.63–2.11)
Overweight (461)	5,689,000	1.86 (0.83–3.55)	1.86 (0.83–3.55)	287,000	106,000	181,000	3.16^¶^ (1.38–5.40)
Obesity (all) (556)	6,726,000	9.43 (6.78–12.97)	3.79 (2.20–6.04)	634,000	255,000	380,000	5.64 (3.66–7.88)
Obesity (severe) (228)	2,705,000	14.70 (10.01–20.51)	5.87 (3.20–9.76)	397,000	159,000	239,000	8.76 (4.68–13.93)
Obesity (not severe) (328)	4,022,000	5.89 (2.91–10.44)	2.38 (0.66–5.96)	237,000	96,000	141,000	3.52 (1.84–5.52)

**Abbreviations:** BMI = body mass index; CI = confidence interval.

* New guideline: adolescents aged 12–17 years were classified as having hypertension if mean systolic or diastolic blood pressure was ≥95th percentile (using 2017 percentile tables), or systolic blood pressure was ≥130 mmHg, or diastolic blood pressure was ≥80 mmHg, or the participant reported were taking antihypertensive medication (available for ages 16–19 years). Persons aged 18–19 years were classified as having hypertension if systolic blood pressure was ≥130 mmHg, or diastolic blood pressure was ≥80 mmHg, or if the participant reported taking antihypertensive medication.

^†^ Former guideline: adolescents aged 12–17 years were classified as having hypertension if mean systolic or diastolic blood pressure was ≥95th percentile (using 2004 age, sex, and height percentile tables), or if the participant reported use of antihypertensive medication. For persons aged 18–19 years, hypertension was defined as systolic blood pressure was ≥140 mmHg, or diastolic blood pressure was ≥90 mmHg, or if the participant reported use of antihypertensive medication.

^§^ BMI is compared with age- and sex-specific reference values from the 2000 CDC growth charts (https://www.cdc.gov/growthcharts/cdc_charts.htm). Healthy = BMI-for-age ≥5th to <85th, overweight = BMI-for-age ≥85th to <95th percentile, obesity = BMI-for-age ≥95th percentile. Severe obesity = BMI-for-age ≥120% of the 95th percentile. Those classified as underweight are excluded.

^¶^ Indicates relative standard error >30%.

## Discussion

According to the criteria of the 2017 AAP Clinical Practice Guideline, approximately one in seven U.S. youths aged 12–19 years had elevated BP or hypertension during 2013–2016. Prevalence of hypertension varied by weight status, ranging from 2% among healthy-weight youths to nearly 14% among those with severe obesity. The new guideline used a lower threshold of hypertension and new percentile references, and compared with the former guideline, the new guideline would reclassify 2.6% of U.S. youths, or nearly an additional 800,000, as having hypertension.

The application of the new guideline results in a net increase in the number of persons aged 12–19 years classified as having hypertension. Early screening (*3*) and intervention should be encouraged. Hypertension among youths is associated with increased risk for hypertension and other markers of cardiovascular risk during adulthood (*1*,*2*); however, if children with hypertension can achieve normal BP by adulthood, this risk might be reduced (*1*). Despite significant increases in the prevalence of obesity and severe obesity from 2001–2004 to 2013–2016, the prevalence of hypertension declined significantly (3.5 percentage points) across this time. This decline in adolescent hypertension is consistent with other reports (*6*,*7*), and might be related to improved diet quality or improved screening and earlier lifestyle or pharmacologic intervention (*8*,*9*). Increases in antihypertensive medication use, and subsequent decreases in BP, might have partially contributed to the observed declines in hypertension. Information on medication use was not available for participants aged 12–15 years and thus could not be included in the definition of hypertension for this age group. In addition, there appeared to be an increase in antihypertensive medication use based on review of the participants’ actual medications, both among youths who self-reported medication use for BP control and were collected in the definition of hypertension, and among youths who did not self-report medication use for BP control and were not included in the definition of hypertension. Although antihypertensive, or BP-lowering, medications are primarily used to manage hypertension, they can also be used for other cardiovascular conditions, migraines, or anxiety. Declines in adolescent hypertension prevalence should be interpreted with caution, as the underlying causes of the decline are uncertain (*7*).

The findings in this report are subject to at least three additional limitations. First, surveys such as NHANES are subject to selection and response bias, which might affect the accuracy of national estimates, despite use of weights and survey procedures. Second, multiple BP measurements were taken on a single day, rather than spread over two or more visits as is recommended for diagnosis (*3*). Finally, self-reported medication use data are subject to recall bias.

Reducing hypertension prevalence among youths is a *Healthy People 2020* objective (HDS-5.2).^¶¶^ Lifestyle interventions for youths with elevated BP or hypertension include increased physical activity and adoption of healthy eating patterns such as the Dietary Approaches to Stop Hypertension (DASH) diet (*3*). Sodium reduction in the food supply and promotion of physical activity in communities and schools are population strategies for improving cardiovascular health (*10*). Pediatricians, family physicians, public health professionals, policy makers, parents, and schools can all be involved in efforts to address hypertension in the adolescent population.

**Summary**

**What is already known about this topic?**

Elevated blood pressure during adolescence is associated with cardiovascular risk in adulthood. In 2017, the American Academy of Pediatrics released a new guideline that changed the criteria for diagnosing hypertension.

**What is added by this report?**

Using the new guideline, an estimated 800,000 additional youths aged 12–19 years (especially older youths, males, and those with obesity) would be reclassified as having hypertension during 2013–2016, compared with using the former guideline.

**What are the implications for public health practice?**

Clinicians and researchers transitioning to the new guideline might expect more youths to be classified as having hypertension. Efforts to address hypertension in youths include lifestyle and environmental strategies that promote cardiovascular health.

## Footnotes

* https://www.nhlbi.nih.gov/files/docs/peds_guidelines_sum.pdf.

† National High Blood Pressure Education Program Working Group on High Blood Pressure in Children and Adolescents. The Fourth Report on the Diagnosis, Evaluation, and Treatment of High Blood Pressure in Children and Adolescents. https://www.nhlbi.nih.gov/files/docs/resources/heart/hbp_ped.pdf.

§ A maximum of three blood pressure readings were measured for each participant in the Mobile Examination Center under a standard protocol. For participants with only a single BP reading, that measurement was used in place of an average. https://wwwn.cdc.gov/nchs/data/nhanes/2013-2014/manuals/Phys_Exam_Manual_2013.pdf.

¶ From NHANES Prescription Medication dataset, unpublished. The definition of hypertension used in this analysis did not include medications identified as being antihypertensives in the Prescription Medication dataset (in which interviewers asked to see all prescription medication containers for medications that the participant had taken within 30 days), because some of these medications might not have been taken for the purpose of controlling hypertension. Hypertension in this analysis only included self-reported antihypertensive use from the Blood Pressure and Cholesterol Module (e.g., “Because of your high blood pressure/hypertension, have you ever been told to take prescribed medicine?”). However, use of medications identified as being antihypertensives in the Prescription Medication dataset increased from 2001 to 2014, the last year for which this dataset was available.

** Guideline for the Prevention, Detection, Evaluation, and Management of High Blood Pressure in Adults. A Report of the American College of Cardiology/American Heart Association Task Force on Clinical Practice Guidelines. http://hyper.ahajournals.org/content/early/2017/11/10/HYP.0000000000000065.

†† https://www.cdc.gov/nccdphp/dnpao/growthcharts/resources/sas.htm.

§§ During 1999–2006, certain groups, including Mexican Americans, were oversampled, while the number of non-Mexican American Hispanics in the NHANES sample was too small for reliable estimates. Because of these sampling differences and sample size concerns, it is not recommended to examine “all Hispanics” from years before 2007. Hispanics other than Mexican Americans were included in the “other” category. https://www.cdc.gov/nchs/data/nhanes/analyticnote_2007-2010.pdf.

¶¶ https://www.healthypeople.gov/2020/topics-objectives/topic/heart-disease-and-stroke/objectives.
